# Ultra-low activities of a common radioisotope for permission-free tracking of a drosophilid fly in its natural habitat

**DOI:** 10.1038/srep36506

**Published:** 2016-11-04

**Authors:** Wolfgang Arthofer, Clemens Decristoforo, Birgit C. Schlick-Steiner, Florian M. Steiner

**Affiliations:** 1 Molecular Ecology Group, Institute of Ecology, University of Innsbruck, Technikerstrasse 25, 6020 Innsbruck, Austria; 2 Department of Nuclear Medicine, Medical University of Innsbruck, Anichstrasse 35, 6020 Innsbruck, Austria

## Abstract

Knowledge of a species’ ecology, including its movement in time and space, is key for many questions in biology and conservation. While numerous tools for tracking larger animals are available, millimetre-sized insects are averse to standard tracking and labelling procedures. Here, we evaluated the applicability of ultra-low, permission-exempt activities of the metastable isomer of the radionuclide Technetium-99 for labelling and field detection of the mountain fly *Drosophila nigrosparsa*. We demonstrate that an activity of less than 10 MBq is sufficient to label dozens of flies and detect single individuals using standard radiation protection monitors. The methodology presented here is applicable to many small-sized, low-mobility animals as well as independent from light and weather conditions and visual contact with the target organism.

Knowledge of a species’ ecology and physiology is a key requisite for conservation management^1^. The temperature shifts of up to 5.3 °C expected for the Alpine area of Central Europe for this century^2^ will likely have massive ecological impacts, including shifts in species distributions, changes in species communities, and, in the extreme case, extinctions. The ‘summit trap phenomenon’^3^ forces species to move with increasing temperatures to higher, that is, cooler, elevations until a mountain’s top is reached and the escape attempt thus stopped. Thermal tolerance of species is commonly assessed by standardised laboratory procedures^4^. However, numerous traits like variance in thermal plasticity^5^,^6^ and behavioural responses^7^ cannot be sufficiently simulated in an artificial environment. To allow sound ecological conclusions, data gathered under field conditions are needed.

For large animals, numerous tools to manually and/or automatically track the movements of an individual in its habitat’s space and time are readily available, for example, direct observation after physical labelling with rings, collars, staining, or tattooing the body surface, automatic camera traps, direct radio tracking, and long-term radio surveillance combined with mobile-phone or satellite based data uplink^8^,^9^,^10^,^11^. More recently, the use of Radio Frequency Identification chips was introduced to tag single individuals^12^,^13^, with the possibility to identify individuals as small as a few centimetres from a distance of several metres. Still, with all these techniques, either size and weight of the labelling devices render their use with millimetre-sized individuals impossible or, in the case of staining and tattooing, close physical contact is required between labelled individual and researcher for successful detection. Furthermore, all of these labelling techniques may alter the behaviour of the labelled individual^14^. Molecular labelling techniques are available for only a few, specialised situations and require extensive wetlab efforts^15^.

The genus *Drosophila*, with more than a thousand species worldwide, (http://www.taxodros.uzh.ch/lists/SPECIES-LIST_GE_SG; retrieved 10 May 2016), harbours some of the best characterised model species in biology^16^. *Drosophila* (*Drosophila*) *nigrosparsa* is a habitat specialist restricted to the European montane/alpine zone^17^ and is currently being established as a study system for testing adaptive evolution under thermal stress^18^. Organisms at high altitudes are considered highly vulnerable to ongoing climate warming^19^ and, due to the summit trap effect, have only a limited possibility to shift to cooler habitats^3^. The possibilities for long-term survival of such a species include rapid evolution and behavioural adaptation^20^,^21^. Alpine habitats often present big temperature differences on small spatial scales^22^, and actively searching for cooler microhabitats might have the potential to compensate for higher average temperatures^23^. To assess insect behaviour in the field, the exact localisation of individuals is required, but the size of *Drosophila* flies prohibits the use of conventional tracking methods.

After the discovery of radioactivity by Henri Becquerel in 1896, radionuclides were extensively used to track animal movement^24^,^25^,^26^,^27^. Radionuclides are easy to administer to the target organism and, at least in the case of β- and γ-radiating isotopes, easy to detect in the field using handheld monitors. However, legal restrictions introduced since the late 1960 s^28^,^29^, the shutdown of many research reactors^30^, and the development of non-radioactive tracking methods as described above, reduced the use of radionuclides over the years.

A radioisotope broadly used for medical applications is the metastable isomer of Technetium-99 (^99m^Tc)^31^. This radionuclide is a pure γ emitter, has a short half-life of six hours, and can be comfortably extracted from radionuclide generators^32^. Standard procedures to introduce ^99m^Tc into tracers with different chemical properties are available^33^,^34^. Today, ^99m^Tc generators can be found in every major hospital^35^ (but see ref. 36). These features render ^99m^Tc an attractive marker for non-medical applications as well (e.g. ref. 37).

Application of radioactive isotopes below the legal exemption levels circumvents the need for an official permit or registration and is common in some applications, for example, their use in luminous paints on wrist watches^38^. For ^99m^Tc, the International Atomic Energy Agency suggests an exemption level of 10 MBq^39^. Austria and many other countries including those of the European Union and Canada have implemented this level; therefore, ^99m^Tc activities below this level are not considered a radioactive substance from a legal point of view (http://www.ris.bka.gv.at/GeltendeFassung.wxe?Abfrage=Bundesnormen&Gesetzesnummer=20004773). Moreover, they still can be easily detected with sensitive radiation probes built for field use. Ultra-low activity amounts of ^99m^Tc thus offer the opportunity for sensitive, permission-free tracers for various biological applications. In this study, we assessed the applicability of ultra-low activity labelling of *D. nigrosparsa* flies using ^99m^Tc as tracer. We addressed the following research questions in detail: (Q1) What are the incorporation rates of three different formulations of the tracer commonly used in nuclear medicine applications? (Q2) Does pre-incorporation starving and dehydration increase tracer uptake? (Q3) Do males and females differ in their incorporation and elimination rates? (Q4) Can ultra-low activity labelled flies be detected efficiently using portable radiation detectors under field conditions?

## Methods

### Preparation and administration of ^99m^Tc fly diet

^99m^Tc was obtained as Na^99m^TcO_4_ (^99m^Tc pertechnetate) from a commercial ^99^Mo/^99m^Tc generator (Ultratechnekow, Mallinckrodt, The Netherlands) eluted with 0.9% NaCl. ^99m^Tc diethylenetriaminepentaacetic acid (^99m^Tc DTPA) and ^99m^Tc human albumin nanocolloid (^99m^Tc nanocolloid) were prepared using Technescan DTPA (Mallinckrodt, The Netherlands) and Nanocoll (GE Healthcare, Germany) kits, respectively, by adding 2000 MBq ^99m^TcO_4_ in 0.9% NaCl following the manufacturer’s instructions. All solutions of pertechnetate, DTPA, and nanocolloid were finally diluted to a concentration of 1200 MBq/ml with 0.9% NaCl.

Grape agar was produced by mixing 42 g red grape juice, 1.7 g sucrose, 1.2 g agar agar, 0.5 g dry yeast, and 25 ml H_2_O. The mixture was gently heated under constant agitation and allowed to boil for 1 min to allow complete dissolution of the agar and inactivation of the yeast cells. Then, 200 μl ^99m^Tc solution (240 MBq) was placed in a 1.5 ml reaction tube (Eppendorf, Germany), 900 μl hot grape agar was added, mixed by pipetting up and down, immediately filled into the lid of a 50 ml centrifuge tube (Sarstedt, Germany), and allowed to solidify.

Ventilation holes were pierced into 50 ml centrifuge tubes using hot insect pins. Batches of 25 flies were anaesthetised lightly using CO_2_ and placed in the tubes, which were closed with lids containing the radiolabelled grape agar. Flies were allowed to feed for one hour at room temperature and ambient light and then either immediately shock frozen in liquid nitrogen or anaesthetised and returned to the malt diet routinely used for maintenance of this species^40^.

### Experiment 1: Effect of tracer formulation and starving/dehydration

For assessing incorporation dynamics (Q1 & Q2), a laboratory-reared line of *D. nigrosparsa* originating from Kaserstattalm (Austria, 47°07′36′′ N, 11°17′30′′ E, 2030 m above sea level, a.s.l.) was used. The line had been in culture for two years on malt medium^40^ at a light:dark period of 16:8 hours, a relative humidity of 70%, and a temperature regime simulating the circadian fluctuations in the Central Alps at 2000 m a.s.l. in August (Table 1; based on W. Schöner, pers. comm.) and the temperatures during fly observations at field baits (own unpublished data) in an MLR 352-H (Panasonic, USA) incubator. In this experiment, only females were used as they are larger, take up more food for egg production, and thus were expected to more readily incorporate the radionuclide. Six batches of 25 two to four week old flies (the use of flies of exactly the same age would have been preferable but was impractical due to species-specific difficulties in culturing *D. nigrosparsa*) were removed from the stock flasks 12 h before exposure to the radiolabelled diet. Three batches were placed on maize agar, while three other batches were placed in empty glass tubes for combined starvation and dehydration. All batches were kept at the conditions described until transfer to the isotope laboratory.

Three ^99m^Tc formulations, pertechnetate, DTPA, and nanocolloid, were offered to both the starved and the nonstarved flies, resulting in six experimental batches. After one hour exposure to the radiolabelled food, flies were killed in liquid nitrogen. From each batch, 20 flies were individually placed in plastic tubes, and activity was measured in a gamma counter (WIZARD^2^, Perkin Elmer, USA). For statistical analyses, we used the raw count per minute (cpm) reads of the gamma counter, corrected for the isotope’s half-life of 6.0067 h. Counting efficiency of the instruments was 45 cpm/Bq. The differences between treatments were analysed by a Wilcoxon-Mann-Whitney-Test in PAST v2.14 41 using α = 0.05 and sequential Bonferroni correction.

### Experiment 2: Sex-specific differences

After confirmation of successful tracer incorporation by females in Experiment 1, sex-specific incorporation differences and the rate of elimination (Q3) were assessed. Two batches of two to four week old female and male lab reared *D. nigrosparsa* from the same line as in Experiment 1 were used. Preparation of the radiolabelled food was as described in Experiment 1, but due to the results achieved just pertechnetate was used as tracer. After one hour exposure to the radiolabelled food, one female and one male batch were killed and measured immediately as described in Experiment 1. The remaining batches were kept on the standard malt diet for 6 h at room temperature and ambient light in the isotope laboratory before killing and measuring. Gamma counter reads were corrected for half-life and analysed as in Experiment 1. Male and female activity were compared with a Fligner-Killeen test of homogeneity of variances in PAST using α = 0.05.

### Experiment 3: Labelling of laboratory-reared flies and detection in the field

**Figure 1:**
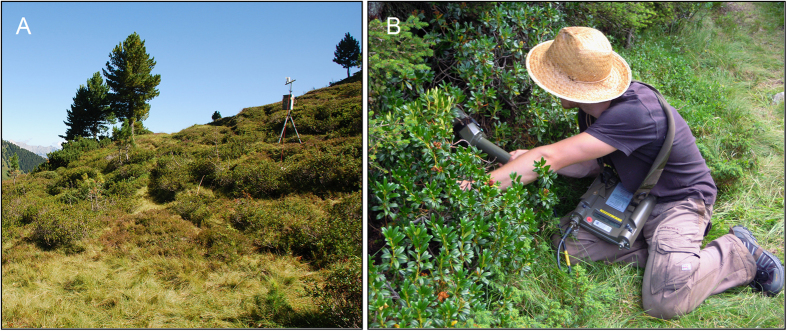
Search for radiolabelled *Drosophila nigrosparsa* flies in their natural habitat. (**A**) Landscape and vegetation at the release site, Wattener Lizum, Austria, 47°11′10′′ N, 11°36′25′′ E, 1972 m a.s.l. (**B**) SSM1 operator searching for elevated gamma radiation (i.e., flies) with the external contamination probe SSM1-12.

On 8 August 2012, a first field experiment was conducted to address Q4. The specific aims were to (i) test the logistics of fly labelling, transport, and release, (ii) evaluate whether detection of single flies and acquisition of environmental data in the field are possible with the instrumentation envisaged, (iii) gain preliminary information on the mobility of the flies after release, and (iv) evaluate the time span after which detection becomes impossible due to radioactive decay, fly dispersal, and/or elimination. It was not intended to produce an ecological dataset in this experiment, and therefore, laboratory-reared flies were used, despite potential biases introduced by their development in an artificial environment; collecting flies in the field for this experiment would have required ca. 200 hours additional workload. Taking into account the lower elimination rate of males (see Results, Experiment 2), and aiming to maximise the detection window shaped by the isotope’s half-life and elimination, we opted here for males rather than females. Thus, ca. 160 male flies were fed pertechnetate as described under Experiment 1. All radiolabelled flies were collected in a 50 ml centrifuge tube (Sarstedt, Germany), and activity was measured in a calibrated ionisation chamber (VDC405, Veenstra, Joure, The Netherlands). As the whole batch of flies exceeded 10 MBq, individual flies were removed, and the measurement was repeated until the radiation officer confirmed the level of radioactivity to be below 10 MBq. From that point, handling of the flies thus required no permit. Within one hour after the end of feeding, the flies were transferred to a potential habitat of the species (Wattener Lizum, Tyrol, Austria, 47°11′10′′ N, 11°36′25′′ E, 1972 m a.s.l., Fig. 1) and released at 11:45 (100 individuals) and 13:00 Central European Summer Time (CEST; 50 individuals) in *Rhododendron ferrugineum* shrub vegetation. To cover the wide variety of microhabitats present in the area of investigation, the two release points were 15 m apart.

Two different instruments were used for field detection: The SSM1 radiation protection measuring instrument combined with the external contamination probe SSM1-12 (Seibersdorf Laboratories, Austria) was used to locate individual flies. This probe is equipped with a 400 cm^3^ organic plastic scintillator (active measuring area 106 cm^2^, energy range 0.03–3.00 MeV). In the laboratory, it was successfully used to detect the signal of single ^99m^Tc-labelled flies against the environmental background from a distance of ca. 1.5 m six hours after isotope feeding. In the field, up to six SSM1 were operated simultaneously. In the study area, high levels of ^137^Cs activity occurred in small, arheic cavities, which were likely contamination remains from the fallout of the 1986 Chernobyl incident. In the first two hours of the experiment, these activities were mistaken for labelled flies, based on SSM1 count rates, but eventually identified as ^137^Cs with a portable gamma spectroscope (MKC-A03, ASPECT, Russian Federation). Afterwards, each putative fly observation was therefore validated spectroscopically.

Fly detection started at 12:00 and ended at 17:00 CEST. To locate individual flies, the SSM1 operators continuously screened an area of 50 × 50 m centred on the midpoint between the two fly release spots by measuring along straight north-south oriented trajectories with ca. 1.5 m distance. Whenever an increase in count rate was observed, the operator followed the source until maximum count rate was achieved. At the source, the potential presence of ^99m^Tc was evaluated by gamma spectroscopy, and time, exact position, and ecological (surface structure, vegetation, exposition to sunlight) and micrometeorological (air and surface temperature, wind speed, global radiation, albedo, precipitation) data were recorded. To retrieve additional information on the mobility of individual flies, the position in the field was flagged with a red plastic clip, and presence or absence of radioactivity was re-assessed in irregular intervals, whenever an SSM1 operator passed by.

### Experiment 4: Labelling of field-captured flies and detection in the field

Because the behavioural traits of laboratory-reared *D. nigrosparsa* may have changed during two years of lab rearing, to obtain an ecological dataset, the experiment was repeated with field-captured *D. nigrosparsa* on 17 August 2012. The flies had been collected from fermented banana baits at Kaserstattalm and Pfitscherjoch (Italy, 46°59′00′′ N, 11°40′35′′ E, 2100 m a.s.l.) two to four weeks before the release date, determined at the species level following the key of^17^, sexed, and kept on standard malt diet until radioactive labelling. Ca. 100 male flies were fed on pertechnetate as described under Experiment 1. Seventy-six male flies with a total activity of 9.5 MBq were released on the same two spots as in the former release experiment at 11:12 CEST (Fig. 1). Continuous detection and acquisition of micrometeorological data as described under Experiment 3 were performed from 11:30 to 18:00 CEST.

## Results

### Experiment 1: Effect of tracer formulation and starving/dehydration

**Figure 2:**
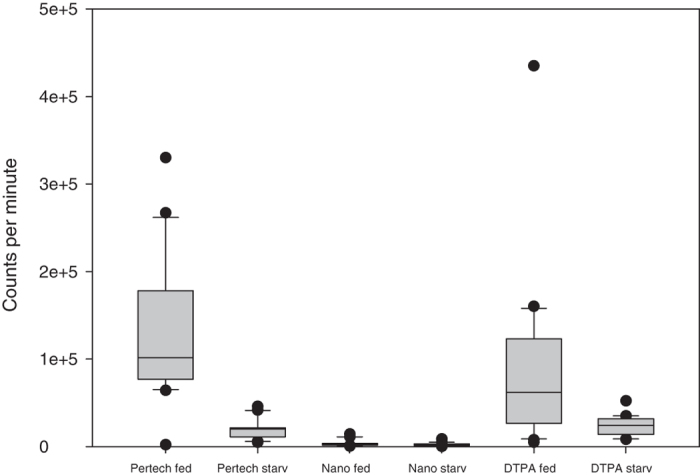
Activity uptake of fed versus starved and dehydrated female *Drosophila nigrosparsa* individuals using three tracer formulations. Insects were either taken directly from culture flasks or initially starved and dehydrated for 12 hours and then allowed to feed on grape agar containing ^99m^Tc for one hour. Unexpectedly, starving significantly reduced subsequent food uptake, probably due to long recovery times after dehydration. Pertechnetate and DTPA are hydrophilic formulations that were easily incorporated by the insects. The non-soluble albumin nanocolloid particles likely bound to solid components of the insect medium preventing activity uptake with sponging mouthparts. Pertechnetate was identified as the best suitable tracer.

The average count rate of fed flies (which corresponds with the activity uptake as described above) was, per fly, 127,583 ± 78,136 (standard deviation) cpm (pertechnetate), 3902 ± 3983 cpm (nanocolloid), and 84,905 ± 94,720 cpm (DTPA). The activity uptake by starved and dehydrated flies was 20,129 ± 11,254 cpm (pertechnetate), 2814 ± 1950 cpm (nanocolloid), and 23,932 ± 11,126 cpm (DTPA) (Fig. 2, Supplementary Table S1). The activity uptake by fed flies on pertechnetate and DTPA was significantly higher than that of the flies in any other treatment (Wilcoxon-Mann-Whitney-Test, P from 2.390·10^–2^ to 2.222·10^−7^, Supplementary Table S1).

### Experiment 2: Sex specific differences

**Figure 3:**
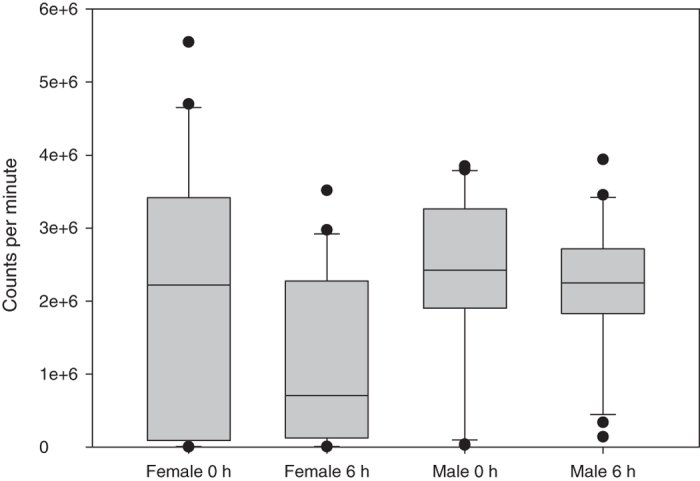
Sex differences in activity uptake and elimination by *Drosophila nigrosparsa*. Male and female individuals of *D. nigrosparsa* were fed for one hour on pertechnetate and killed and measured either immediately or six hours after feeding. The results were corrected for the half-life of the isotope. While no significant sex bias in activity uptake was detected, the average activity in females compared with males was significantly lower after six hours, suggesting faster elimination due to higher metabolic rate and/or egg laying. Large standard deviations in all treatments were caused by the presence of single flies not taking up any activity, that is, not feeding during the one-hour exposition to the labelled food. Non-feeding individuals were significantly more often female than male.

The average count rate of female flies was, per fly, 1,041,643 ± 872,350 cpm, that of male flies 1,201,094 ± 574,416 cpm (Fig. 3, Supplementary Table S2). The high standard deviations were caused by individual flies that did not incorporate the isotope (Supplementary Table S2), that is, did not feed during the 1-hour exposition to the radiolabelled grape agar. Uptake did not differ between the two sexes (Wilcoxon-Mann-Whitney-Test, P = 0.3507). After six hours on non-radioactive malt diet, the average count rate, corrected for the isotope’s half-life, was 598,169 ± 571,164 cpm for females and 1,105,069 ± 454,375 cpm for males. Thus, the elimination rate in females was almost 50%, while males showed almost no elimination; this difference was highly significant (Wilcoxon-Mann-Whitney-Test, P = 0.0084). Furthermore, while non-feeding individuals occurred in both sexes, the portion of non-feeders was higher in females (Fligner-Killeen test of homogeneity of variances; CV_female_ = 92.89, CV_male_ = 44.53, P = 0.0001). Directly comparing the zero- and six-hour activities within sex was not possible as the flies had to be killed for the measurements, that is, the populations at the two points in time were not identical.

### Experiment 3: Field detection of laboratory-reared flies

Labelling flies with an activity sufficiently low to not be regulated by Austrian radiation protection law was unproblematic, as were transport to and release in the field. The on-site background count rate of the SSM1 ranged from 83 to 125 cpm, and the rate increased to >200 cpm when a single labelled fly was placed 100 cm in front of the detector at the time of release. From 11:45 to ca. 14:00 hours CEST, numerous spots with elevated count rates (200–500 cpm) were recorded as fly observations. As these observations were always located near the ground and, in repeated measurements, never changed location and activity, assessment by gamma spectroscopy was applied. ^137^Cs was identified as activity source; all measurements prior to this were discarded and, in the following, each SSM-1-based detection of elevated activity was evaluated with the gamma spectroscope. The first spectroscopically verified detection of a fly occurred at 15:00 in *Rhododendron* vegetation. Subsequently, 13 verified fly encounters were made, the last one at 16:25 when the experiment was terminated.

### Experiment 4: Field detection of field-captured flies

Successful detections of released flies occurred between 44 and 360 minutes after their release (Supplementary Table S3). In total, 26 locations with ^99m^Tc activity were identified and flagged. In three instances, a living fly was visually detected after activity-based localization. Assuming that each location represented a single fly, 34.2% of the released flies were detected. The minimum distance travelled by individually detected flies was 0.3 m, the maximum distance 11.0 m. When reassessing previously flagged locations at a later point in time, the radionuclide was still present in 13 instances, indicating that these flies had not moved away; at one location, activity was detected at three points in time. The time between first and last detection was between 38 and 157 minutes, indicating that the involved individuals did not move for at least these time periods. At 10 spots where activity had been detected, we verified absence of activity in a subsequent measurement between 48 and 225 minutes after the first detection.

## Discussion

Tracking individuals in the field is essential for the assessment of many ecological and behavioural traits but difficult in species with small body size to which classical tracking such as using radio transmitters is not applicable. Drosophilid flies are smaller than 5 mm and weigh less than 5 mg, and are thus not traceable with conventional methods. In this study, we investigated the feasibility of ultra-low activity radioactive labelling to track individual *D. nigrosparsa* flies in the field for several hours.

^99m^Tc is a radionuclide commonly used in medical diagnosis^31^. Due to its short half-life of six hours, it is usually produced on-site in radionuclide generators, from which it is eluted as pertechnetate ion and then either directly administered to patients or immediately further processed into various radiopharmaceuticals^32^,^33^. In humans, pertechnetate enriches in the thyroid gland and the stomach while the highly hydrophilic DTPA is quickly eliminated through the kidneys. Non-soluble nanocolloid particles have the slowest elimination rate in humans.

Adding the isotope to a common insect medium, grape agar, and allowing the flies to feed on this medium for one hour was an efficient method of labelling, with three notable observations (Fig. 2, Supplementary Table S1). First, both pertechnetate and DTPA resulted in high isotope uptake. In contrast, the albumin-nanocolloid formulation was unsuitable for fly labelling. When separating the liquid from the solid phase of the medium via centrifugation, we observed that radioactivity was present in the liquid phase of both the pertechnetate and DTPA supplemented grape agar but not in that of the albumin nanocolloid. We suspect that the nanocolloid binds strongly to the solid components of the grape agar and thus becomes unavailable for *Drosophila*, due to its sponging mouthparts. Insects with chewing mouthparts might have produced different results in this experiment. Second, in all treatments, pronounced differences in activity uptake were observed among individuals, resulting in high standard deviations. This result is in line with observations from *D. melanogaster*^42^. Furthermore, adult *D. melanogaster* flies spend only about 10% of their time feeding^43^, and when assuming a similar behaviour for *D. nigrosparsa*, a feeding period of one hour is probably too short to allow homogeneous food uptake by the whole population. While longer feeding might alleviate this effect, it would be impractical when using ^99m^Tc due to the short half-life of the isotope. In our case, the presence of individuals with little to none incorporation was unproblematic, as we were aiming at a sufficient number of individuals with high uptake and not at a homogeneous activity over the whole population. Third and unexpectedly, starving and dehydrating the flies for 12 hours prior to exposition to the radioactive diet did not improve activity uptake but, in contrast, massively reduced the uptake even of the otherwise efficient formulations pertechnetate and DTPA. At the time of the experiment, little experience with suitable starvation conditions was available for *D. nigrosparsa*. Various *Drosophila* species are reported to tolerate desiccation under low relative humidity conditions between 5 and 35 hours^44^, but recovery from dehydration can be a lengthy process^45^. Probably, 12 hours without a water supply put *D. nigrosparsa* close to its physiological margins, and one hour feeding on the labelled diet was too short for most flies to recover from dehydration stress.

Two factors define the time span radiolabelled individuals remain traceable: (i) the half-life of the isotope used and (ii) the elimination of the tracer. While (i) is a physical property of the isotope beyond control, we expected sex based differences in (ii). Female drosophilid flies often show higher food uptake than males^46^,^47^ but also increased metabolic rates and elimination due to egg laying^48^. While the average isotope uptake immediately after the end of exposure to the labelled grape agar did not differ between males and females (P = 0.3507, Fig. 3, Supplementary Table S2), the activity in males was higher than in females six hours after exposure (P = 0.0084). This effect was caused by a sharp decrease of average activity in the female population while the activity in the males decreased only slightly. Thus, faster elimination rather than unequal feeding amounts seems the driver of sex bias in labelling.

When performing the final release experiment to create an ecological dataset, only males were used to maximise the detection window in the field. In the case of sex dependent behavioural differences (e.g., increased flight activity of males compared with females), this bias must be taken into account when interpreting the results from localising individuals in the field. Furthermore, this release experiment was performed using field-collected flies, but to collect sufficient numbers, maintenance of the flies for a maximum of four weeks in captivity was unavoidable. To our knowledge, effects of such short-time captivity on fly behaviour have not been studied, and it remains unclear whether this treatment influenced our results.

It is also unknown to which degree radiation itself influences fly behaviour. The male fly with the highest individual activity seen in Experiment 2 had 1,954,407 cpm immediately after labelling (Supplementary Table S2), corresponding to 42,765 Bq. This fly would have accumulated a dose of 5.17 mSv in the ten hours of our field experiment and 7.55 mSv in ten half-lifes of the isotope, a period after which radioactivity can be considered as completely decayed (see Supplementary Table S4 for a detailed calculation). This dose is higher than the average background radiation in Austria (2.8 mSv/year;^49^). On the other hand, the threshold for the onset of acute radiation syndrome in humans is 500 to 1000 mSv within a few hours^50^, and insects are known to be even more radiation tolerant: in a recent study on Sterile Insect Technique^51^, pupae of *Aedes albopictus* survived a dose of 35,000 mSv. We thus consider the direct influence of radiation in our experiment as negligible.

A critical procedure before releasing the labelled flies into the environment was the reliable quantification of the activity incorporated. While during media preparation and feeding, ^99m^Tc activities of ca. 250 MBq were used for each batch of 25 flies (for a thyroid scintigraphy, typically 75 MBq are administered per patient), and thus an isotope laboratory was required, the activity of the whole fly batch to be released had to be below 10 MBq to stay below legal limits. This activity can easily be quantified with ionisation chambers (“dose calibrators”) routinely used in Nuclear Medicine departments to measure the activity in syringes for patient applications.

This study provides a proof of principle that the ultra-low activity labelling of small, low-mobility insects and subsequent detection in the field are feasible, with a detection rate of 34.2% of all released flies. For 10 observations (13.2% of all flies) where an initially detected activity disappeared, we can deduce that the flies were alive at the time of the first detection and moved away later. For other species to be labelled with ^99m^Tc, the methodological details should be optimised anew, including a suitable food source, suitable activity under consideration of hourly food uptake and body size, and elimination. More detailed study of incorporation and elimination, and/or individual labelling and tracking might help overcome the presence of non-feeders and diminish the high standard deviations observed in Experiment 2. Another way, not assessed in the frame of this study, would be to administer the isotope to the body surface, for example, by dusting or spraying. While this would warrant for a more even labelling among individuals, the isotope might be lost by, for instance, grooming or contact with wet surfaces. In any case, the trade-off between complexity of experimental setup and homogeneity of labelling has to be considered for each new application of this technique.

Compared with other labelling and tracking techniques, ^99m^Tc offers several advantages: Body size is not limiting, and even much smaller organisms than *D. nigrosparsa* could be labelled by just increasing the specific radioactivity of the food. As no direct, visual observation is necessary, the method performs equally well in daylight and in darkness. Detection is possible under any weather condition and not hampered by animals’ camouflage. As the γ radiation of this isotope is quite penetrative, for example, even subterranean individuals might be detectable.

The main limitation of ultra-low activity ^99m^Tc labelling is the rather short window for detection of six to nine hours, after which the radioactive decay will cause the loss of a detectable signal. Thus, the method is not suitable for long-term observation and problematic in cases where the distance between the radionuclide generator laboratory and the area of investigation is long. Phosphorous-32 (^32^P), an isotope with a half-life of 14.28 days, was broadly used in biological research in former decades and still has some niche applications. It has been successfully used for fly labelling^24^ and might be an alternative to overcome the short half-life of ^99m^Tc. However, the legal exemption limit of ^32^P is 100 kBq, which seems too low for most ecological applications. Exceeding this limit would imply costly permissions and mandatory radiation protection measurements.

It should also be mentioned that the technical equipment for this technique is expensive. Anyway, ^99m^Tc generators are operated at practically all larger hospitals, and detection equipment is often available from civil and military emergency response organizations like police, fire brigades, and CBRN (chemical, biological, radiological, nuclear defence) teams. Establishing cooperation with emergency response organizations will, for the scientists, provide substantial cost savings and transfer of knowledge in field detection and, for the organizations, often be a welcome opportunity to practice with their equipment.

We consider ultra-low activity labelling with ^99m^Tc a very suitable approach for permission-free field tracking of small, low-mobility individuals where no other suitable techniques are available. Potential applications may include, among others, ecological monitoring, detection of preferred microhabitats in the context of conservation or climate change, and tracking of otherwise not observable organisms like wood boring or subterranean insects.

## Additional Information

**How to cite this article**: Arthofer, W. *et al*. Ultra-low activities of a common radioisotope for permission-free tracking of a drosophilid fly in its natural habitat. *Sci. Rep.* **6**, 36506; doi: 10.1038/srep36506 (2016).

**Publisher’s note:** Springer Nature remains neutral with regard to jurisdictional claims in published maps and institutional affiliations.

## Supplementary Material

Supplementary Information

## Figures and Tables

**Table 1:** Temperature regime used in environmental test chambers for cultivation of *Drosophila nigrosparsa.*

Daytime [hh:mm]	Temperature [°C]	Light
23:00–05:00	7	no
05:00–11:00	10	yes
11:00–17:00	14	yes
17:00–18:00	25	yes
18:00–19:00	23	yes
19:00–20:00	21	yes
20:00–21:00	19	yes
21:00–23:00	10	no
